# Sex differences in the evolution of left ventricle remodeling in rats with severe volume overload

**DOI:** 10.1186/s12872-020-01360-0

**Published:** 2020-02-03

**Authors:** Elisabeth Walsh-Wilkinson, Marie-Claude Drolet, Marie Arsenault, Jacques Couet

**Affiliations:** Groupe de recherche en valvulopathies, Centre de recherche de l’Institut universitaire de cardiologie et de pneumologie de Québec, Université Laval, 2725, Chemin Sainte-Foy, Québec City, G1V 4G5 Canada

**Keywords:** Cardiac hypertrophy, Echocardiography, Sex dimorphism, Rats, Volume overload, Left ventricle, Aortic regurgitation

## Abstract

**Background:**

Aortic valve regurgitation (AR) results in left ventricle (LV) volume overload (VO) leading to its dilation and hypertrophy (H). We study a rat model of severe AR induced by puncturing one or two leaflets using a catheter. Most of our studies were conducted in male animals. Recently, we started investigating if sex dimorphism existed in the AR rat model. We observed that AR females developed as much LVH as males but morphological remodeling differences were present. A head-to-head comparison of LV morphological and functional changes had never been performed in AR males (M) and females (F) using the latest modalities in cardiac imaging by echocardiography.

**Methods:**

We performed a longitudinal study to evaluate the development of LV hypertrophy caused by chronic AR in male and female rats over 6 months. Sham-operated (sham) animals were used as controls.

**Results:**

LV diastolic volumes (EDV) increased more over 6 months in sham males than in females (38% vs. 23% for EDV, both *p* < 0.01). AR resulted in significant LV dilation for both sexes (54% vs. 51% increase in EDV) vs. baseline values. Since normal cardiac growth was less in females, dilation from AR was relatively more important for them (88% (M) vs. 157% (F) increase in EDV over sham). AR caused LV wall thickening in both males and females. It happened sooner for AR females and was more important than in males (25% (M) vs. 56% (F) increase in septum thickness at 2 months and 10% (M) vs. 30% (F) at 6 months). We then evaluated if AR was associated with changes in LV strain using speckle-tracking 2D echocardiography. Global longitudinal strain remained similar between AR and sham animals. Circumferential strain was negatively modulated by AR but only in females and early after VO induction (13% (M) vs. 26% (F)).

**Conclusion:**

AR resulted in more LV dilation and quicker wall thickening in female AR rats compared to males. Global circumferential strain was negatively modulated in AR females but not in males. AR also seemed to lead to a more spherical LV shape in females whereas; it kept mostly an ellipsoid shape in males. This can influence validity of mass estimation of the dilated LV in females by echocardiography.

## Background

Study of volume overload (VO) has sometimes lagged behind the research dedicated to pressure overload (PO). Often secondary to other causes, heart valve regurgita­tion has received less interest in part because their main historical cause, rheumatic fever, has been mostly eradicated in modern industrialized countries. Rheumatic valve diseases are still occurring at a significant rate in low/middle-income countries and in poor and remote indigenous communities in richer countries. The estimated burden worldwide of rheumatic valve diseases is estimated to more than 15 million existing cases with 280 k new cases each year and 230 k deaths [1]. One main cause of secondary aortic valve regurgitation (AR) is related to valve replacements in patients with aortic stenosis. For instance, a small proportion of patients (5–10%) undergoing transcatheter aortic valve replacement (TAVR) display moderate to severe AR [2]. Since TAVR is now a procedure routinely performed, management of secondary AR is a developing concern. Mitral regurgitation is also a relatively com-mon consequence of non-revascularized cardiac ischemia. The myocardial response to VO from heart valve regurgitation is less well understood and there is a need for more pre-clinical research to address this issue.

Women represent over lifetime 50% of heart failure (HF) patients. It is now well recognized that clinical presentation and risk factors for HF present sex dimor­phisms. HF is often more ischemic in men, happens sooner in life and leads to re­duced left ventricle (LV) ejection fraction (EF). In women, HF happens later in life, is more often a consequence of a lingering hypertensive disease and EF, preserved [3, 4]. Female patients with aortic valve stenosis (a PO disease) usually develop more LVH but have better EF and less myocardial fibrosis than male patients [5]. More left ventricle hypertrophy is also observed in hypertensive women compared to men even for similar blood pressure [6]. Sex dimorphisms are also well-described in pre-clinical models of HF. In mice with transverse aortic constriction (TAC; a LV PO model), males develop concentric LVH sooner than females and evolve more rapidly towards eccentric LVH and HF with reduced ejection fraction (HFrEF) [5]. A sex dimorphism is thus present in the hypertrophic response to PO in both pa­tients (hypertension) and in pre-clinical models (TAC) [7]. If sexually dimorphic LV response to overload has been relatively well-documented in PO situations, this is not the case for VO.

We have studied for a number of years, LV remodeling in response to significant VO from experimental AR in male Wistar rats. We observed that it resulted in important LV dilation in order to accommodate the excess of regurgitating aortic blood [8, 9]. In this rat model of chronic VO, we observed that female animals developed as much if not more LVH than males over 6 months [10]. Males displayed more eccentric LVH (dilation to wall thickening ratio) and worse EF than females. On the other hand, AR females had more LV wall thickening compared to males [10, 11]. Progression towards HFrEF is not a feature of the AR rat model. In another rat VO model (aorto-caval fistula), a faster progression toward HFrEF was observed in males and resulted in poorer survival compared to females [12].

In this study, we wished to document LVH development in the AR rat model and identify morphological and functional sex differences. New capacities in cardiac imaging by echocardiography (echo) allow better characterization of morphological changes taking place during LVH development. The use of four-dimensional (4D) analysis can provide new information about LV geometry and volumes, whereas strain analyses using speckle-tracking two-dimensional (2D) echo can help detect early dysfunction. We thus longitudinally studied by echo, male and female AR rats over a 6-month period. Healthy sham-operated rats of both sexes were used as controls.

Our results indicates that LV VO from AR may be more adverse for females than suggested by our past studies [10, 11, 13]. LV dilation is relatively more important compared to sham controls and systolic function is quickly diminished after AR induction in females compared to males. LV geometry is also different between AR male and female animals.

## Methods

### Animals

Rats were purchased from Charles River (St-Laurent, Canada). Severe AR was induced in males (300-325 g, *n* = 14/group) and females (200-225 g, n = 14/group) Wistar rats (9–10 weeks of age) by retrograde puncture of one or two aortic valve leaflets under echocardiographic guidance as previously described [8, 9]. Only animals with more than 50% of regurgitation were included in the study. Several animals (2 for each sex) were excluded from the follow-up on this basis. Sham-operated rats had their right carotid ligated (*n* = 8/group). Rats were randomly assigned to the sham or AR group. The regurgitant fraction was estimated by the ratio of the forward sys­tolic .ow time–velocity integral (VTI) to the reversed diastolic flow VTI measured by pulsed Doppler in the thoracic descending aorta. Animals were housed in pairs in standard plastic caged (same experimental group). Fibercore was used for bedding; a red plastic tunnel and chewing toy were provided for environmental enrichment. The protocol was approved by the Université Laval’s Animal Protection Committee and followed the recommendations of the Canadian Council on Laboratory Animal Care.

### Echocardiography

Echo studies: Echo studies were performed the day before AR surgery, then 2 weeks, 2 months, 4 months post-operation and at the end of protocol (6 months). Echo images were acquired using Vevo LAB software on a Vevo 3100 imaging system (VisualSonics, FujiFilm, Toronto, Canada) by the same investigator. The investigator was blinded for animal identification but it was not possible to do so for the different groups (sex and AR). Rats were anesthetized and positioned on a platform ventral side up. The concentration of isoflurane was maintained around 2–2.5%, so the heart rate was between 350 and 370 beats/minute.

2D echo: M-mode images were recorded to measure diastolic and systolic LV wall thickness from the parasternal long-axis (PSLAX) view and the short-axis (SAX) view at the papillary muscle level. From these measurements, LV Mass was obtained using the following equation: 1.053 x [(EDD + PW + IVSW)^3^ - EDD^3^] where: EDD is the internal dimension of the LV at the end of diastole, PW is the thickness of the posterior wall at the end of diastole and IVSW is the thickness of the inter-ventricular septum at the end of diastole [14, 15]. A corrected LV mass was also calculated by the VevoLab echo analysis software (VisualSonics) corresponding to the estimated LV mass from the equation above multiplied by 0.8. Ejection fraction from M-mode images was also calculated using the following equation: (EDD^2^ - ESD^2^)/EDD^2^ where: ESD is the internal dimension of the LV at the end of systole. Pulsed wave Doppler was used to measure the mitral flow from an apical four-chamber view. Peak early diastolic filling velocity (E wave), peak filling velocity at atrial contraction (A wave), E wave deceleration slope (slope) and the E/A ratio were calculated.

4D echo: 4D-Mode images were acquired from the PSLAX and short-axis (SAX; at the papillary muscle level) views to measure LV diastolic and systolic volumes. 4D-Mode is a 3-dimensional EKV mode (ECG-based Kilohertz Visualization) image acquisition at every 3D motor position during a complete heart cycle. Thus, we obtained a 2D loop at every motor position, creating a 4D clip of the heart cycle. The Multi-slice method was used to measure LV volumes. LV contour was drawn for 3 slices minimum (middle and both LV end) in the PSLAX and SAX views. Vevo LAB software automatically determined contour on every slice between the drawn contours. These contours were manually adjusted, if needed. These steps were completed for the first, the middle and the quarter time point. The volume is displayed in mm^3^ for every slice and every time point. Diastolic volume, systolic volume, stoke volume (SV; Diastolic volume-Systolic volume) and ejection fraction (EF; 100 x ((Stroke volume)/(Diastolic volume))) were obtained from these LV volume measurements.

Speckle-tracking echo (STE): 2D echo B-mode loops were acquired from the PSLAX and SAX views and analysed using Vevo Strain software (VisualSonics). Images were acquired at the highest frame rate possible and strain analysis was performed in the longitudinal, radial and circumferential axes. PSLAX view was used for longitudinal and radial strains, whereas SAX view was used for circum­ferential strain analysis. Three cardiac cycles of the highest quality cine loop were selected to avoid animal respiration, echo gel artefact, and significant obstruction from the ribs. Endocardial and epicardial borders were traced at mid-diastole. For the PSLAX view, tracing was started from the anterior wall close from the aorta root to the posterior wall close from the mitral valve. For the SAX view, borders were traced in the counter-clockwise direction, starting from the top anterior wall. Vevo Strain software then build the dynamic LV tracing for all selected frames. Cine loops were replayed to confirm good border tracking over cardiac cycles and manual adjustments were made if needed. LV myocardium was divided into 6 equal anatomical segments and peak systolic strain were calculated for every segment. Peak systolic strain = (Ls - Ld)/(Ld) where: Ls = Length at end-systole and Ld = Length at end-diastole. Global peak systolic strain in the 3 directions were calculated by averaging the peak systolic values of the 6 segments.

At the end of the protocol, euthanasia was performed under isoflurane anesthesia. Saturated potassium chloride (2–3 ml) was injected directly in the heart. The thorax was then opened and the heart and lungs were harvested and weighed.

### Statistical analysis

Results are presented as the mean and the standard error of the mean (SEM). Statistical analyses were performed on the log of the data. (Graph Pad Prism 8.02, San Diego, CA). A Student’s t-test was used when two groups were compared. ANOVA and Holm-Sidak post-test was used when more than two groups were compared. A *p* value lower than 0.05 was considered significant. Raw data from this study are presented as an Additional file 1.

## Results

### Animal characteristics

Eleven out of twelve AR rats were alive at the end of the protocol for each sex. All sham-operated rats survived the duration of the protocol. In Table 1 are summarized the characteristics of the animals at the end of the protocol. As expected, AR caused important increases in total heart weight as well as for the left ventricle, right ventricle and left atrium weight. The increase in heart weight was similar for both male and female AR rats compared to sham (around 70%). AR similarly increased LV weight in males and females (72% increase in males vs. 76% for females). The same was true for the right ventricle (32% vs. 35%) and left atrium (174% vs. 164%). Indexed values of heart and left ventricle weights increased accordingly.

**Table 1:** Characteristics of sham-operated and AR animals at the end of the protocol

Parameters	ShM (*n* = 8)	ARM (*n* = 11)	ShF (n = 8)	ARF (n = 11)
Body weight, g	800 ± 27	790 ± 25	480 ± 24	441 ± 22
Tibial length, mm	61 ± 0.3	61 ± 0.3	53 ± 0.2	53 ± 0.3
Heart, mg	1626 ± 45	2777 ± 114*	1059 ± 26	1801 ± 78*
Heart/BW, mg/g	2.1 ± 0.05	3.5 ± 0.10*	2.2 ± 0.08	4.2 ± 0.22*
Left ventricle, mg	1226 ± 37	2112 ± 67*	796 ± 20	1399 ± 52*
LV/BW, mg/g	1.5 ± 0.04	2.7 ± 0.08*	1.7 ± 0.07	3.2 ± 0.17*
Right ventricle, mg	300 ± 12	405 ± 20*	197 ± 7	261 ± 19*
Left atria, mg	39 ± 4	107 ± 12*	22 ± 1	58 ± 9*
Lungs, g	2.7 ± 0.18	3.0 ± 0.17	2.0 ± 0.10	2.1 ± 0.09

*BW* body weight and *LV* left ventricle. Values are expressed as the mean ± SEM. Group comparisons were made using two-way ANOVA followed by Holm-Sidak post-test. *: *p* < 0.0001 vs. respective sham group

### Echocardiography data

AR (Table 2) significantly changed most echo parameters. End-diastolic and end-systolic LV diameters measured by M-mode, were both increased in AR males and females. On the other hand, LV walls (septal; SW and posterior; PW) were significantly thicker compared to respective sham groups in females but not in males. Diastolic function parameters such as the E and A waves and E wave slope were unchanged in AR rats compared to sham after 6 months but also compared to baseline values measured 6 months earlier (Table 3).

**Table 2:** Echocardiographic parameters (short-axis view, M-mode images) of male and females animals at the end of the protocol

Parameters	ShM (n = 8)	ARM (n = 11)	ShF (n = 8)	ARF (*n* = 10)
AR severity, %	NA	66 ± 5	NA	73 ± 4
EDD, mm	9.4 ± 0.2	12.6 ± 0.3*	7.9 ± 0.1	11.1 ± 0.3*§
iEDD, mm/kg	11.8 ± 0.4	16.1 ± 0.6*	16.6 ± 0.7	26.3 ± 1.4*§
ESD, mm	5.4 ± 0.3	8.4 ± 0.4*	4.3 ± 0.1	7.5 ± 0.3*
iESD, mm/kg	6.8 ± 0.4	10.6 ± 0.6*	9.0 ± 0.1	17.6 ± 1.1*§
IVSW, mm	1.6 ± 0.02	1.6 ± 0.03	1.2 ± 0.03	1.5 ± 0.03*
PW, mm	1.9 ± 0.06	2.3 ± 0.06	1.6 ± 0.05	2.1 ± 0.05*
RWT, unitless	0.36 ± 0.011	0.32 ± 0.006*	0.36 ± 0.010	0.33 ± 0.010*
EF, %	70 ± 2	60 ± 3*	79 ± 1	59 ± 2*
E wave, cm/s	89 ± 2	88 ± 4	80 ± 3	81 ± 4
A wave, cm/s	49 ± 3	41 ± 2	39 ± 1	39 ± 3
E wave slope	4278 ± 402 &	4185 ± 355	3954 ± 246	4626 ± 371

*NA* non applicable, *EDD* end-diastolic diameter, *ESD* end-systolic diameter, *i* indexed for body weight (kg), *SW* septum wall thickness, *PW* posterior wall thickness, *RWT* relative wall thickness, *HR* heart rate, *bpm* beats per minute. Values are expressed as the mean ± SEM. Group comparisons were made using two-way ANOVA followed by Holm-Sidak post-test. *: *p* < 0.05 vs. respective sham group. §: *p* < 0.05 between AR groups

**Table 3:** Echocardiographic parameters (short-axis view, M-mode images) of male and female Wistar rats at baseline (10–11 weeks of age)

Parameters	Males (*n* = 22)	Females (n = 22)
EDD, mm	7.9 ± 0.1	7.0 ± 0.1
ESD, mm	4.4 ± 0.1	3.6 ± 0.1
IVSW, mm	0.8 ± 0.03	0.7 ± 0.03
PW, mm	1.2 ± 0.04	1.1 ± 0.03
LV mass, mg	389 ± 18	318 ± 16
RWT, unitless	0.24 ± 0.009	0.26 ± 0.008
EF, %	74 ± 1.2	81 ± 1.3
E wave, cm/s	85 ± 2.9	85 ± 3.6
A wave, cm/s	52 ± 2.8	50 ± 2.2
E wave slope	3728 ± 197	3993 ± 200

*EDD* end-diastolic diameter, *ESD* end-systolic diameter, *SW* septum wall thickness, *PW* posterior wall thickness, *LV* left ventricle, *RWT* relative wall thickness, *FS* Fractional shortening. Values are expressed as the mean ± SEM

**Fig. 1:**
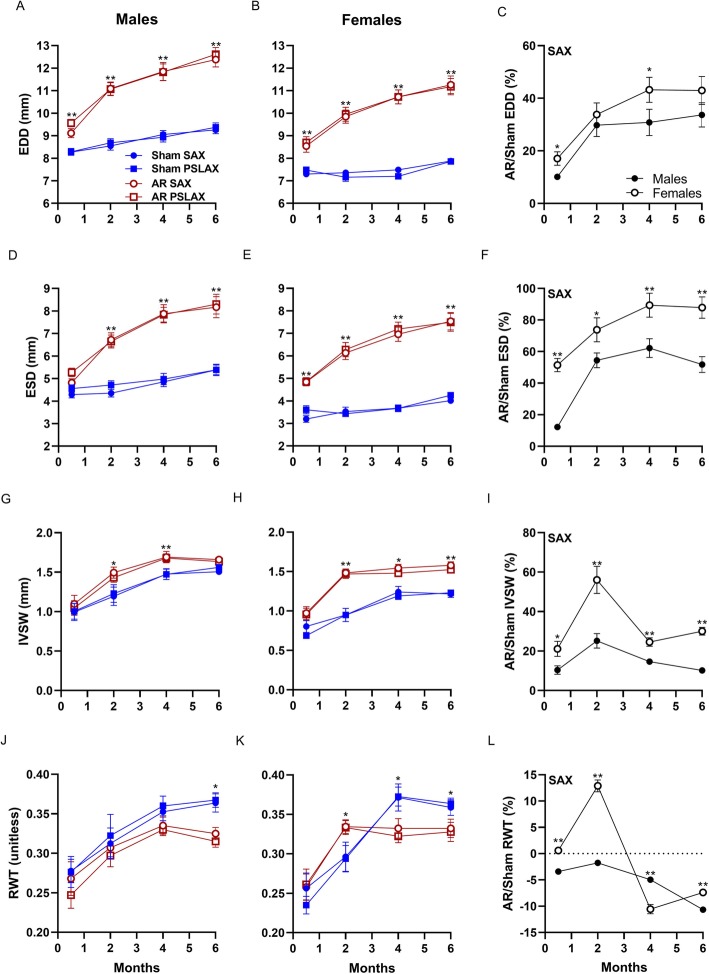
Left ventricular dimensions increases more strongly in female AR rats than in males compared to sham-operated animals. End-diastolic (EDD; **a** and **b**), end-systolic (ESD; **d** and **e**) LV diameters and septum thickness (IVSW; **g** and **h**) were measured in M-mode LV short axis (SAX) and parasternal long axis (PSLAX) views in sham (blue) and AR (red) male and female rats at four different time points over a 6 months after surgery. Relative wall thickness (RWT; **j** and **k**) was then calculated using the equation described in the Methods section. Ratios of the mean values for each parameter in AR rats over sham ones was calculated for each time point and are illustrated in Panels **c**, **f**, **i** and **l**. Results are expressed as mean ± standard error of the mean (SEM; *N* = 8–10 animals/group). *: *p* < 0.05 and **: *p* < 0.01 between corresponding sham and AR groups at a given time point

We used two views to estimate 2D echo parameters namely PSLAX and SAX views. As illustrated in Fig. 1, both views provide similar LV measurements. In sham animals, over the 6 months of the study, normal cardiac growth was more important in males. LV EDD remained stable in females whereas in males, the increase was steady. As expected, AR caused important increases in LV diameters. This was more important for females than for males and more evident for the ESD (Fig. 1a-f). Inter-ventricular septal wall thickness (IVSW) increased in both sham and AR animals resulting in a raise of the relative wall thickness (RWT) during the protocol. In AR animals, increase in IVSW thickness was more important in females than in males (Fig. 1g-i). As expected for VO, RWT decreased, suggesting eccentric LV remodeling compared to sham animals in both male and female AR rats. Interestingly, LV wall thickening taking place early in AR females first resulted in concentric LV remodeling then reversed with increasing LV dilation to a reduced RWT (Fig. 1j-l).

**Fig. 2:**
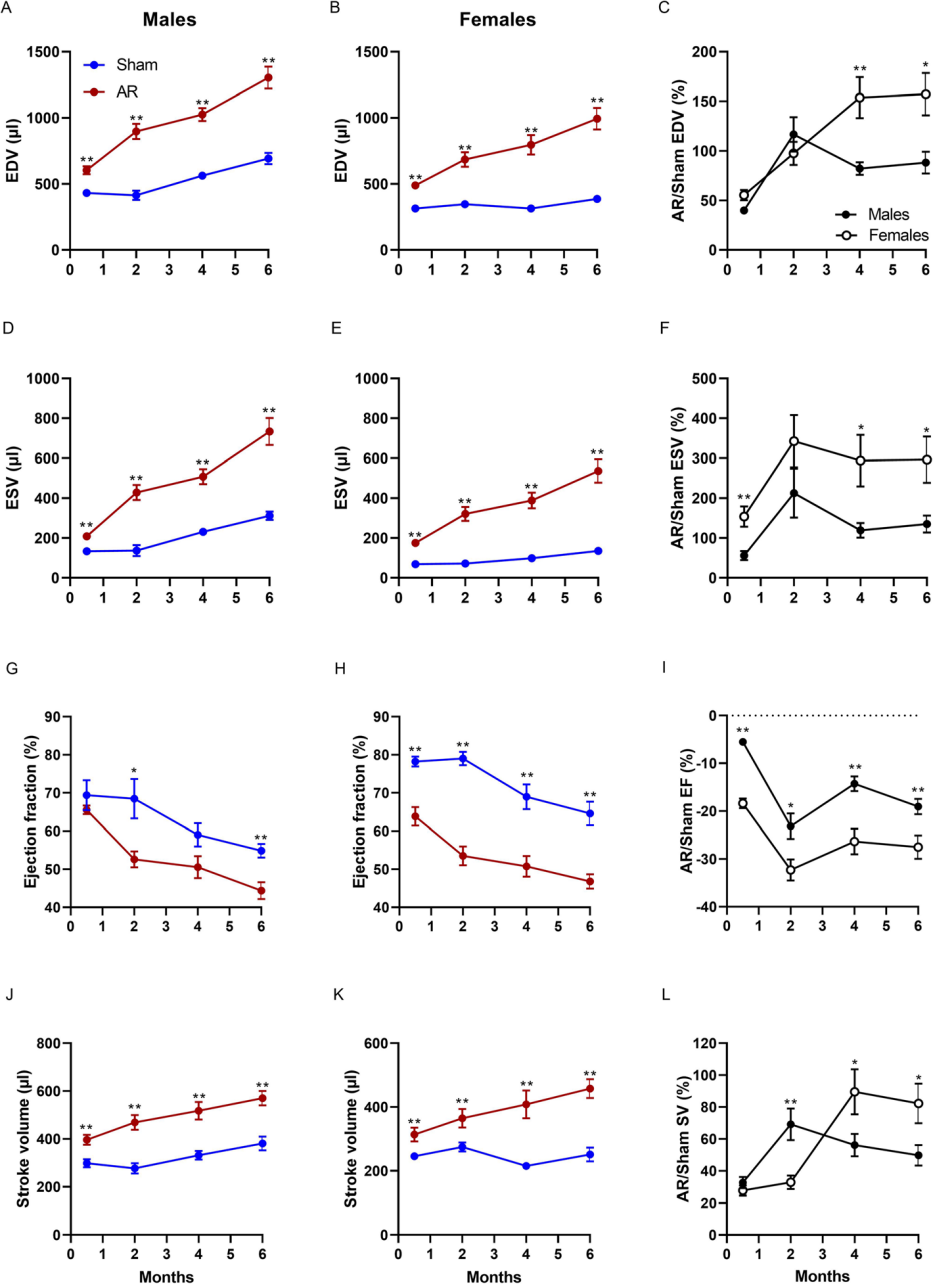
Left ventricular volumes increase more strongly in female AR rats than in males compared to sham-operated animals. End-diastolic (EDV; **a** and **b**) and end-systolic (ESV; **d** and **e**) LV volumes were estimated using three-dimensional reconstruction of stacked LV short axis (SAX) views in sham (blue) and AR (red) male and female rats at four different time points over a 6 months after surgery. LV ejection fraction (EF; **g** and **h**) stroke volume (SV; **j** and **k**) were then calculated using the equation described in the Methods section. Ratios of the mean values for each parameter in AR rats over sham ones was calculated for each time point and are illustrated in Panels **c**, **f**, **i** and **l**. Results are expressed as mean ± standard error of the mean (SEM; *N* = 8–10 animals/group). *: *p* < 0.05 and **: *p* < 0.01 between corresponding sham and AR groups at a given time point

Four dimensional (4D)-mode images were acquired from the PSLAX and SAX views to measure LV diastolic and systolic volumes. In Fig. 2, only results obtained using SAX views are illustrated in order to avoid overloading graphs. End­diastolic volumes (EDV) in sham animals increased by 60% in males but only by 22% in females suggesting that normal cardiac growth was more limited in females during the course of the protocol. AR caused strong increases in EDV as expected. These resulted in AR females EDV being more than 2.5 times larger than sham ones from 4 months after surgery whereas AR males EDV were about 1.9 times larger than sham (Fig. 2a-c). End-systolic volumes (ESV) followed a similar trend being larger in AR females than in males compared to respective sham group after 6 months (296% vs. 135%) (Fig. 2d-e). LV ejection fraction (EF) decreased over 6 months for both sham and AR animals. This decrease was more pronounced for AR animals and appeared as soon as 2 weeks after surgery in females. The loss of EF in AR animals compared to sham was relatively more important for females although final values were similar for both AR groups (Fig. 2g-i). Stroke volume (SV) remained stable over 6 months in sham females while it raised in males. In AR rats, SV increased throughout the protocol to reach 1.5 time values recorded in sham males and 1.8 time for females (Fig. 2j-l).

**Fig. 3:**
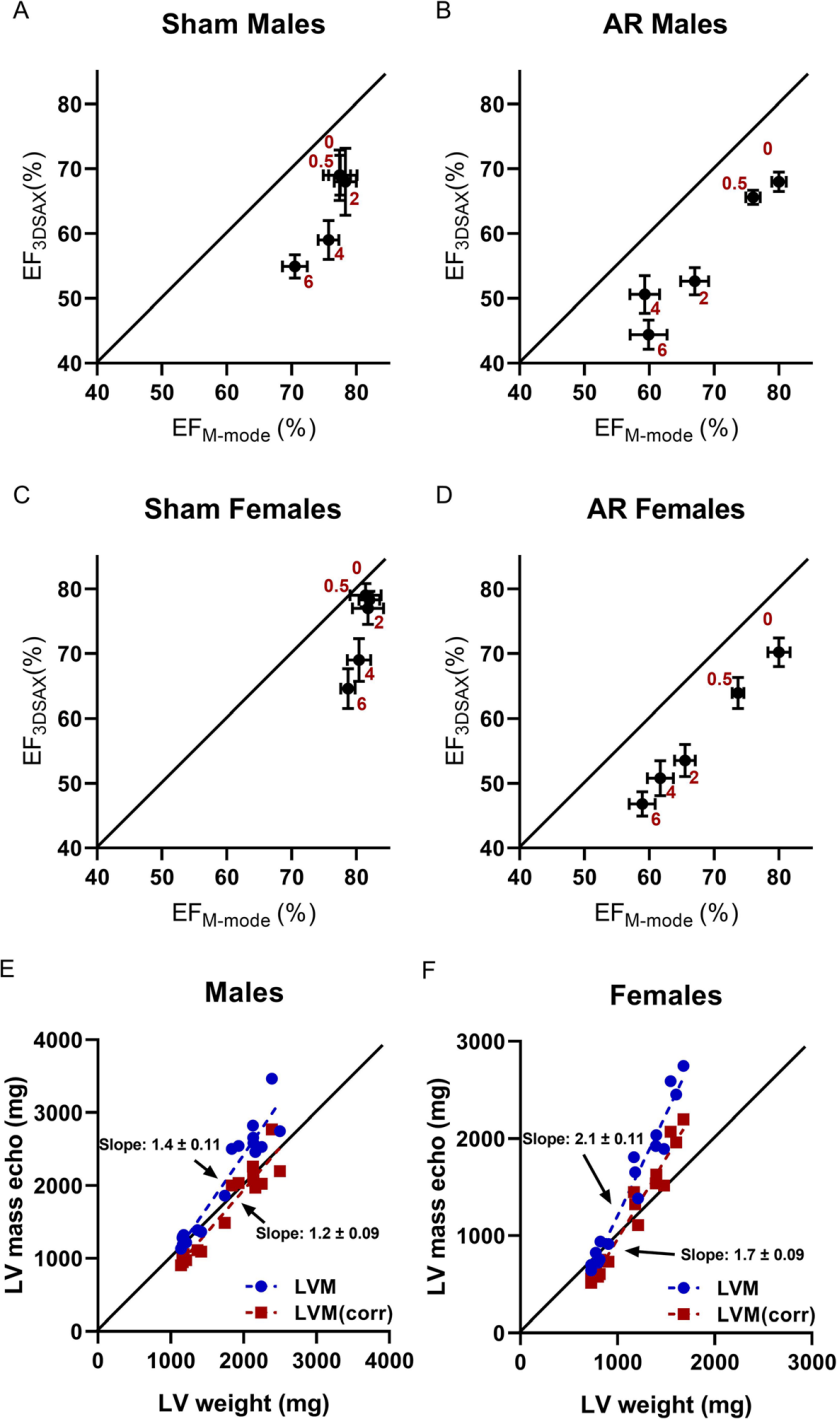
Comparison of various echo methods to estimate ejection fraction and LV mass. Means ±SEM of calculated ejection fractions either using M-mode SAX view (X-axis) or three-dimensional reconstruction of stacked LV short axis (3DSAX) views (Y-axis) were plotted for each time point (red; months) for sham (**a** and **c**) and AR (**b** and **d**) groups. The oblique solid line represents the expected results if both methods were equivalent. In panels **e** and **f** are shown the correlation of estimated LV mass by echo (Y-axis) compared to wet LV weights obtained at the end of the protocol at 6 months. Two equations described in the Methods section were used to estimate LV mass in blue and red, respectively. Slope ± SEM for each regression line is indicated. The oblique solid line represents the expected results if methods were equivalent

**Fig. 4:**
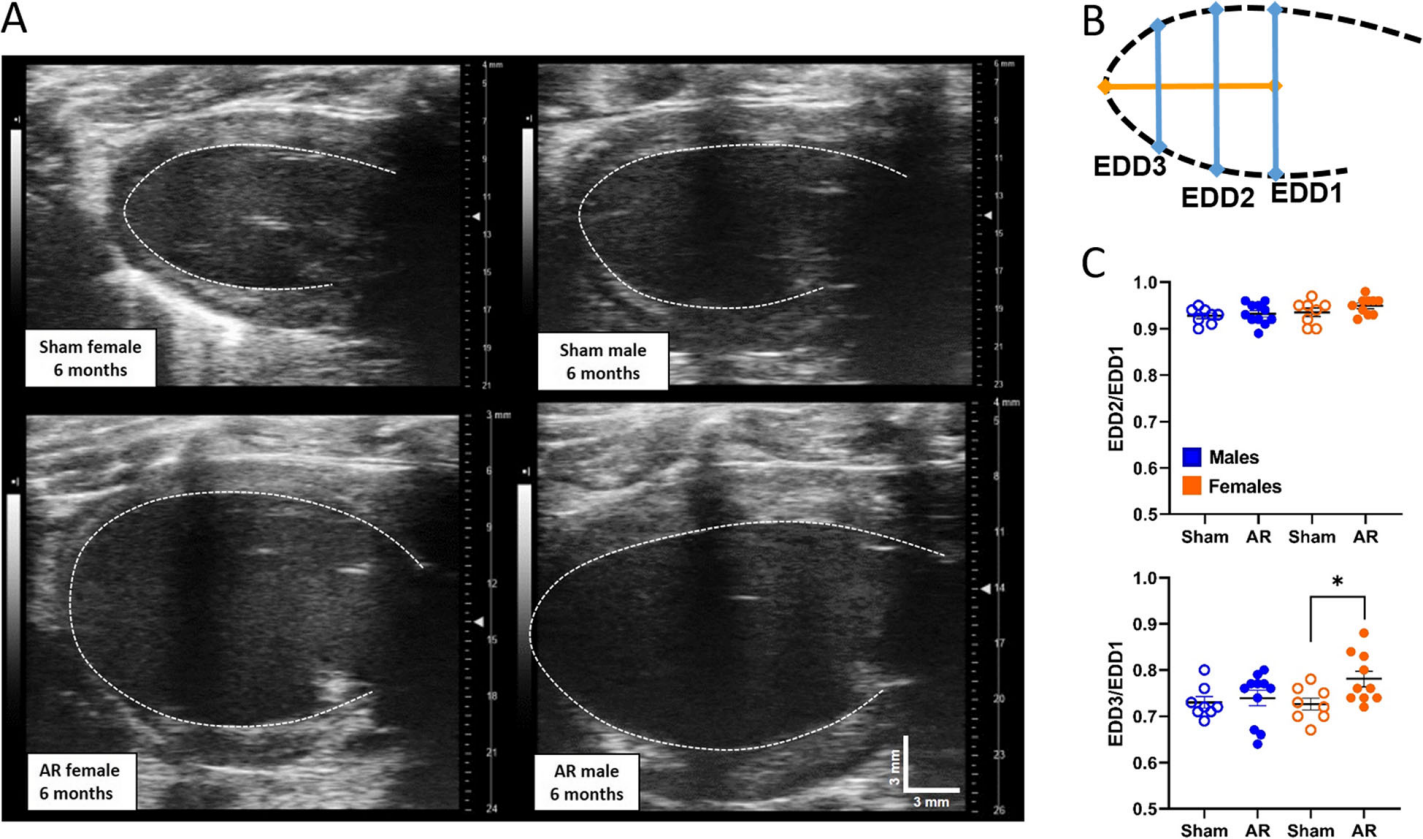
Evidence of a sex dimorphism in the LV geometry of the dilated AR left ventricle. **a**. Representative end-diastolic parasternal long axis (PSLAX) LV views from 6-month sham (up) and AR (bottom) rats of both sexes (left: females and right: males). Images were calibrated in order to be at the exact same scale. Vertical and horizontal bars correspond to 3 mm. **b**. From the PSLAX LV end-diastolic views at 6 months of sham and AR rats, three diameters were determined (EDD1 to 3). EDD1 represents the normal EDD from data or Table 2. The bottom part of the LV was then divided into thirds along the longitudinal axis (orange line) and EDD2 and EDD3 were determined. **c**. Ratio of EDD2/EDD1 (top) and EDD3/EDD1 (bottom) were plotted for male and female sham and AR animals at 6 months. Results are expressed as mean ± standard error of the mean (SEM; *N* = 8–11 animals/group). *: *p* < 0.05 between indicated groups

The estimation of ejection fraction using M-mode 2D views (Table 2) and 4D­mode images (Fig. 2g-i) lead to marked differences in estimated values. In Fig. 3, we plotted the mean ± standard error of EF values for both methods at each time point of the protocol for sham (A and B) and AR (C and D) rats. Using M-mode views for the estimation of EF led to higher values for every group at every time points. Interestingly, EF values calculated from M-mode views were less able to discriminate changes occurring with aging in sham animals. We were then interested to correlate LV mass estimation using echo compared dissected LV weight. In males, both echo methods correlated well with values from the weighted tissue. For females, larger LV mass as found in AR animals were overestimated by echo equations (Fig. 3e-f). LV mass echo equations infer that this heart chamber has an ellipsoid shape. As illustrated in Fig. 4a, general shape of the end-diastolic LV inner border tracing in sham animals and AR males is indeed one of an ellipse. It does not seem to be the case for AR female LVs, which become more spheroid. In order to substantiate this observation, we measured the end-diastolic LV diameter on PSLAX view at three different locations. The standard LV EDD measurement at the level of the papillary muscle was designated EDD1. We then measured LV length from EDD1 to the apex. Two other EDD measurements (EDD2 and EDD3) were then performed respectively at one and two third of this length as indicated in Fig. 4b. Ratios of EDD2 and EDD3 on EDD1 diameter were then calculated. As illustrated in Fig. 4c, the EDD2/EDD1 ratio remained similar between the groups in both AR males and females compared to sham controls although important LV dilation had taken place in AR rats. The EDD3/EDD1 ratio, on the other hand was significantly more elevated in AR females compared to sham. This ratio remained similar in sham animals and AR males.

**Fig. 5:**
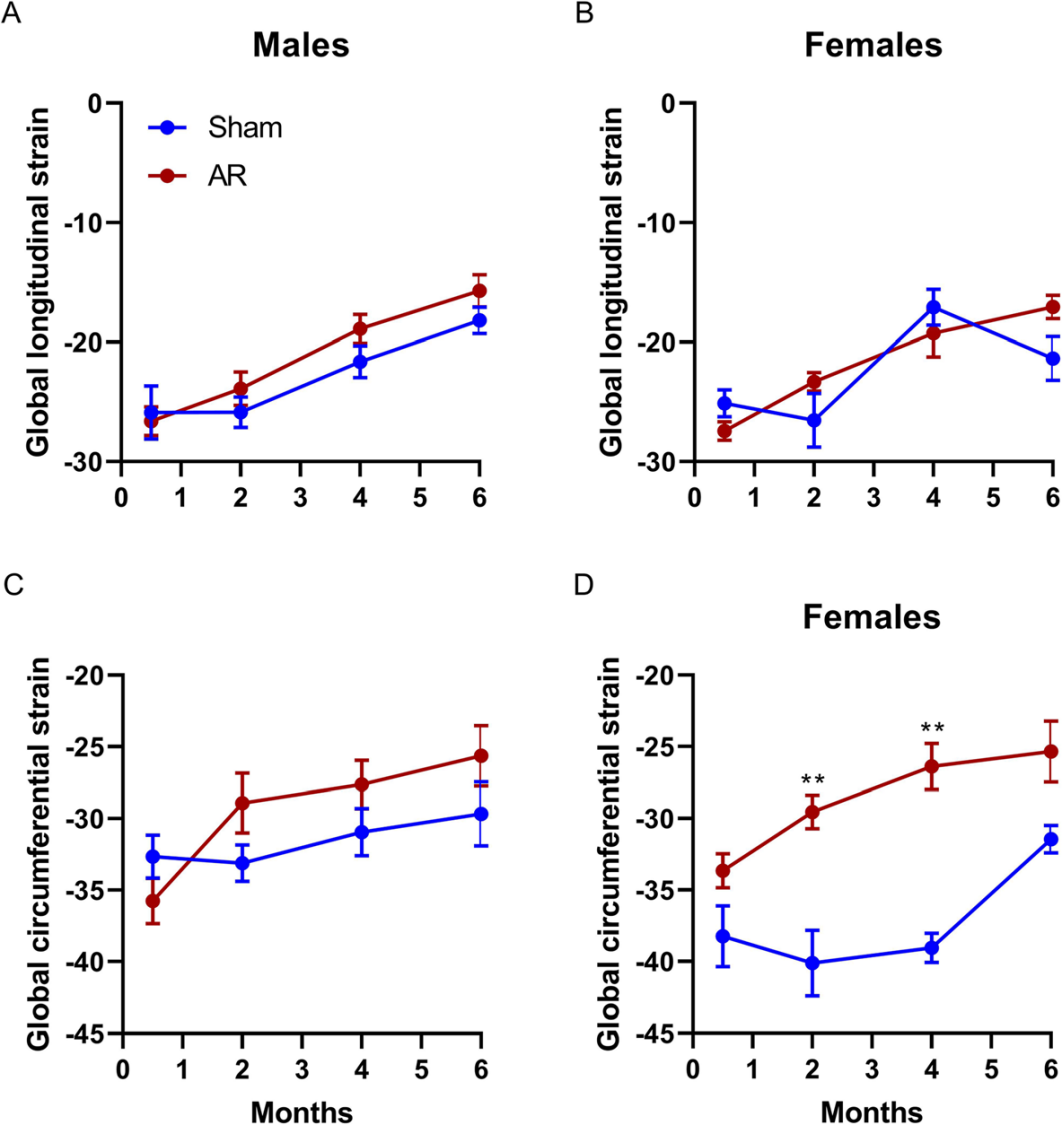
Evolution of global LV strain rates in male and female sham and AR rats. Global longitudinal (**a** and **b**) and circumferential (**c** and **d**) strain rates were calculated from parasternal long axis LV views in sham (blue) and AR (red) male and female rats at four different time points over a 6 months after surgery. Results are expressed as mean ± standard error of the mean (SEM; N = 8–10 animals/group). **: *p* < 0.01 between corresponding sham and AR groups at a given time point

In Fig. 5 are illustrated the evolution of global longitudinal (GLS) and global circumferential (GCS) strains in male and female sham and AR rats. Strain is defined as the fractional change in a dimension in comparison to the original dimension. For both parameters, GLS and GCS, values that are more negative are associated with better fractional change of the myocardium during the cardiac cycle. In sham animals, both GLS and GCS remained stable for the first 2 months before dete­riorating slightly, afterwards (Fig. 5a-d). This deterioration took longer in sham females for GCS, becoming evident at 4 months (Fig. 5d). In AR animals, strain values also became worse with time as expected. Interestingly, GCS strain values were only significantly different between sham and AR animals in females.

## Discussion

The availability of improved pre-clinical imaging systems designed for small rodents now allow thorough analysis of the geometric and functional changes taking place during the development of cardiac remodeling and can help identify differences associated with the biological sex. In this study, we observed several differences in the evolution of LV remodeling in male and female rats where a severe and similar aortic valve regurgitation had been induced. AR females displayed relative to sham control more dilation and wall thickening, quicker decrease in ejection fraction as well as deterioration of global circumferential strain compared to AR males. Diastolic function remained similar between sham and AR animals notwithstanding the biological sex.

In both males and females, LV end-diastolic volume gained 50% over their baseline values after only two weeks of severe VO. This raise in LV volume stabilized after 2 months in males (85% over sham), and after 4 months in females (150% over sham). In sham females, LV EDV and stroke volume (SV) remained mostly stable (+ 23% and + 2%, respectively) over time. In males, EDV and SV increased over time in sham males (+ 60% and + 28%, respectively). This suggests that normal cardiac growth taking place in females did not resulted in the LV pumping more blood in sham females although end-diastolic diameter was larger (Tables 2 and 3). Ejection fraction went down by about 10% in sham rats of both sexes. It is interesting to put these SV values in parallel with the changes of body weight taking place during the protocol. Male and female body weights more than doubled over 6 months whereas cardiac output increased by 37% in males and remained stable in females (− 2.4%). This suggests that the animals, which were sedentary, adapted their cardiac physiology accordingly and the gain of mass (mostly fat) did not result in an increase in circulatory needs. Our results also underscore the need of proper negative sham controls with similar age and obviously, of the same biological sex to put correctly in perspective changes caused by a cardiac overload.

Observations made in this study may lead to the conclusion that LV hypertrophy from AR is less well tolerated by female rats. LV dilation was more important in females compared to males in spite of similar severity of AR levels. Loss of EF came earlier for females although at the end of the protocol, EF values were similar for all AR animals. This seems to go against observations we had reported in the past comparing AR males and females [10]. We had also observed previously that LV dilation and wall thickening were more important in AR females compared to males but loss of EF was less for AR females than in males. In addition, we had shown that myocardial capillary density was significantly reduced in AR males but not in females. We speculated that this would help the LV in AR females maintaining a better access to both oxygen and nutriments for cardiomyocytes. Moreover, we demonstrated that most genes related to oxidation of fatty acids (the preferred energy substrate of the myocardium were more down-regulated in AR males than in females probably forcing the AR male myocardium to rely more on glucose as an energy substrate [10]. Hypertrophy markers were expressed similarly between the sexes, though.

Two-dimensional echo parameters were very similar between this study and the ones presented here [10]. It is possible that the geometry of LV remodeling is being at play in the sex differences observed here. Results obtained in the present study suggests that in females, both LV dilation, significant wall thickening and loss of global circumferential strain characterize early response to AR. It is important to note that GCS was better in females than males at baseline. A similar observation had been reported in young women having a more important GCS compared to men [16]. It has been shown that women have greater LV twist mechanics than males during acute reductions to preload [17, 18]. Here, in a situation of acute increase of preload after AR induction, it is possible that the female LV twist mechanics maybe more strongly affected for females. Early LV dilation in AR females resulted in GCS values similar to males. LV dilation in females probably leads to a more spheroid shape. In fact, it appeared the apex region of the LV in AR females underwent a marked geometric change whereas in males, the general shape of the LV was kept more elliptic. We were not able to con.rm. this using 3D reconstructions of LVs mostly because of interference caused by the animal ribs.

LV ejection fraction values obtained by more conventional methods using 2D echo, seemed to be overestimated compared to those coming from 4D echo as illustrated in Fig. 3. We had reported in the past using micro-positron emission tomography (micro-PET) scan, EDV and ESV from LV 3D reconstructions from male sham and AR rats [19]. Using micro-PET scan, we obtained LV volumes that were similar to those reported here (around 10% of difference) in sham males. For AR males, LV volumes obtained by micro-PET scan were higher (25–30%) than those we obtained here. Rats had similar age in both studies. Normal ejection fraction was estimated as a little over 50% and in AR rats, EF was around 40%, values similar to those obtained here using 4D echo [19]. 2D echo data in these two studies differed by less than 5%. We also observed 2D echo underestimated the loss of EF probably taking place in sham animals (Fig. 3a-c). This was not the case for AR animals. EF was probably overestimated by 2D echo but followed a similar trend as the one obtained from 4D echo. Again, changes in LV geometry taking place during normal cardiac growth of sedentary rats may be difficult to observe when limited to measurements using 2D M-mode echo.

Role of sex steroids in the control of LV remodeling and development of HF are well recognized. In the AR rat model, we recently their contribution in the hyper­trophic response to severe VO. In male rats, loss of testosterone by orchiectomy (Ocx) reduced normal cardiac growth of both sham and AR rats and pathological LVH in AR animals [20]. For females, loss of estrogens also resulted in lesser cardiac growth but it did not significantly modulate the hypertrophic response to AR [21]. The roles of estrogens in the development of LVH has been studied mostly in PO animal models. Less attention was given in VO situations such as in valve regurgitation models or in the aorto-caval fistula (ACF) model. ACF females develop less hypertrophy than males and evolve more slowly towards HFrEF. Their survival is also better [22]. This advantage is in part, dependent on estrogens presence as ovariectomy (Ovx) worsen disease development in females [23]. Estradiol treatment can reverse the effects of Ovx [24]. Results reported here and in recent studies [11, 20], in the AR rat model seem to contradict the observations made in the ACF rat model. ACF primarily is a more global stress that affects the right heart and lungs whereas AR cause a more direct LV stress. In the ACF model studies, male rats evolved towards HFrEF whereas in the AR model, overt HF seldom happens [12, 24]. Most deaths in the AR model are sudden, happen during the active period of the day (night) and are probably caused by malign arrhythmia [19, 25, 26].

We want to point out several limitations in this study. Heart adaptations to VO in women (with the exception of pregnancy) have received very little attention in the literature. Observations made in animals cannot necessarily be transposed to humans, and caution must be used. This study did not take into account ageing (only young rats were used) and menopause (Ovx), factors that are highly relevant to heart disease in women. Simpson’s method was not used for the evaluation of EF as routinely performed in humans. We limited ourselves to the simple 2D M-mode method which is the most frequently used in rodents and the one using volumes estimated from 4D echo. Longitudinal strain could not be integrated from three-angle views: apical four chamber, two chamber and long-axis views, because rat anatomy limits good quality image acquisition in apical views.

## Conclusion

In conclusion, we showed that sex differences exist between male and female rats in which a severe LV VO was induced. Both the extent of the hypertrophic response to AR and its development showed interesting sexual dimorphisms.

## Supplementary information

**Additional file 1.** Raw data of the study.

## Data Availability

All data generated or analysed during this study are included in this published article and its additional files.

## Abbreviations

2D: Two-dimensional
3D: Three-dimensional
4D: Four-dimensional
ACF: Aorto­caval fistula
ANGII: Angiotensin II
ANOVA: Analysis of variance
ANP: Atrial natriuretic peptide
AR: Aortic regurgitation
CH: Cardiac hypertrophy
D: Diastole
ECG: Electrocardiogram
EDD: End-diastolic diameter
EDV: End-diastolic volume
EF: Ejection fraction
EKV: ECG-gated kilohertz visualization
ESD: End­systolic diameter
ESV: End-systolic volume
GCS: Global circumferencial strain
GLS: Global longitudinal strain
HF: Heart failure
HFrEF: Heart failure with reduced ejection fraction
IVSW: Interventricular septal wall
L: Length
LV: Left ventricle
LVH: Left ventricle hypertrophy
OVX: Ovariectomized
PET: Positron emission tomography
PO: Pressure overload
PSLAX: Parasternal long axis
PW: Posterior wall
RAAS: Renin-angiotensin II-aldosterone system
RWT: Relative wall thickness
S: Systole
SAX: Short axis
SEM: Standard error of the mean
SV: Stroke volume
TAC: Transverse aortic constriction
TAVR: Transcatheter aortic valve re­placement
VO: Volume overload
VTI: Flow time-velocity integral
